# Toner Lectures

**Published:** 1873-06

**Authors:** 


*Toner Lectures.*

A series of lectures has been instituted at Washington under the "Toner Fund to encourage the discovery of new truths for the advancement of Medicine." The trustees of the fund are the following distinguished gentlemen: Joseph Henry, LL.D., Secretary Smithsonian Institution; Joseph K. Barnes, M.D., Surgeon-General, U.S.A.; J. C. Palmer, M.D., Surgeon-General, U.S.N.; James E. Morgan, M.D., President Medical Society, D. C.; J. M. Toner, M.D., President American Medical Association.

The first lecture of this course was delivered at Washington, by Dr. J. J. Woodward, U. S. A., March 28th, upon the subject of "The Structure of Cancerous Tumors, and the manner in which adjacent parts are invaded." The lecture was illustrated by some seventy photographic screen illustrations, and was successful. The second of the course was given by Dr. C. E. Brown-Sequard, his subject being "Nervous Force; the extent, variety and power of its manifestations."

